# Targeting viral entry as a strategy for broad-spectrum antivirals

**DOI:** 10.12688/f1000research.19694.1

**Published:** 2019-09-12

**Authors:** Michela Mazzon, Mark Marsh

**Affiliations:** 1 MRC Laboratory for Molecular Cell Biology, University College London, London, UK

**Keywords:** Antiviral, Virus entry, Endocytosis, Virus fusion, Broad-spectrum, Host-targeted

## Abstract

The process of entry into a host cell is a key step in the life cycle of most viruses. In recent years, there has been a significant increase in our understanding of the routes and mechanisms of entry for a number of these viruses. This has led to the development of novel broad-spectrum antiviral approaches that target host cell proteins and pathways, in addition to strategies focused on individual viruses or virus families. Here we consider a number of these approaches and their broad-spectrum potential.

## Introduction

Although not a guarantee of successful infection, entry into a host cell is a critical stage in the life of a virus ^1, 2^. Once inside a cell, a virus still needs to overcome a hostile environment, evade immune responses, and subvert a range of cellular proteins and pathways to facilitate its replication. However, finding a permissive cell and delivering genetic information into its cytoplasm, and in some cases nucleus, is the first necessary step for viral infection. This has at least two important implications from a prophylactic and/or therapeutic perspective. The first is that blocking viral entry stops infection early on, preventing viral replication. The second is that because many viruses exploit cellular endocytic mechanisms to initiate internalization and infection, and cells have just a few such mechanisms, inhibiting these pathways may affect many different viruses, greatly expanding our currently limited antiviral portfolio.

It is now well established that, after initial attachment to the cell surface, many viruses, both enveloped and non-enveloped, exploit changes in environmental conditions, such as pH, interaction with a cellular receptor, or the activity of proteolytic enzymes, to trigger conformational changes in key proteins that mediate cell membrane penetration ^1– 3^. For enveloped viruses, the virus lipid bilayer needs to fuse with the cellular limiting membrane (either the plasma membrane or the membrane of endocytic organelles) to release the viral genome, and this is mediated by viral envelope proteins. For non-enveloped viruses, genome release is affected by viral capsid proteins that trigger cell membrane penetration and genome release.

While some viruses fuse directly at the plasma membrane, the majority use endocytic mechanisms to reach intracellular compartments, with clathrin-mediated endocytosis (CME), caveolin-mediated endocytosis, macropinocytosis, and phagocytosis currently being the best characterized of these pathways. CME in particular is exploited by numerous viruses, including alphaviruses, flaviviruses, orthomyxoviruses, and rhabdoviruses ^1, 2^, while macropinocytosis is linked to the uptake of larger viruses, such as poxviruses and filoviruses, that do not fit into clathrin-coated vesicles ^4– 6^. Thus, inhibiting these pathways might be expected to impact on a range of different viruses.

**Figure 1.**
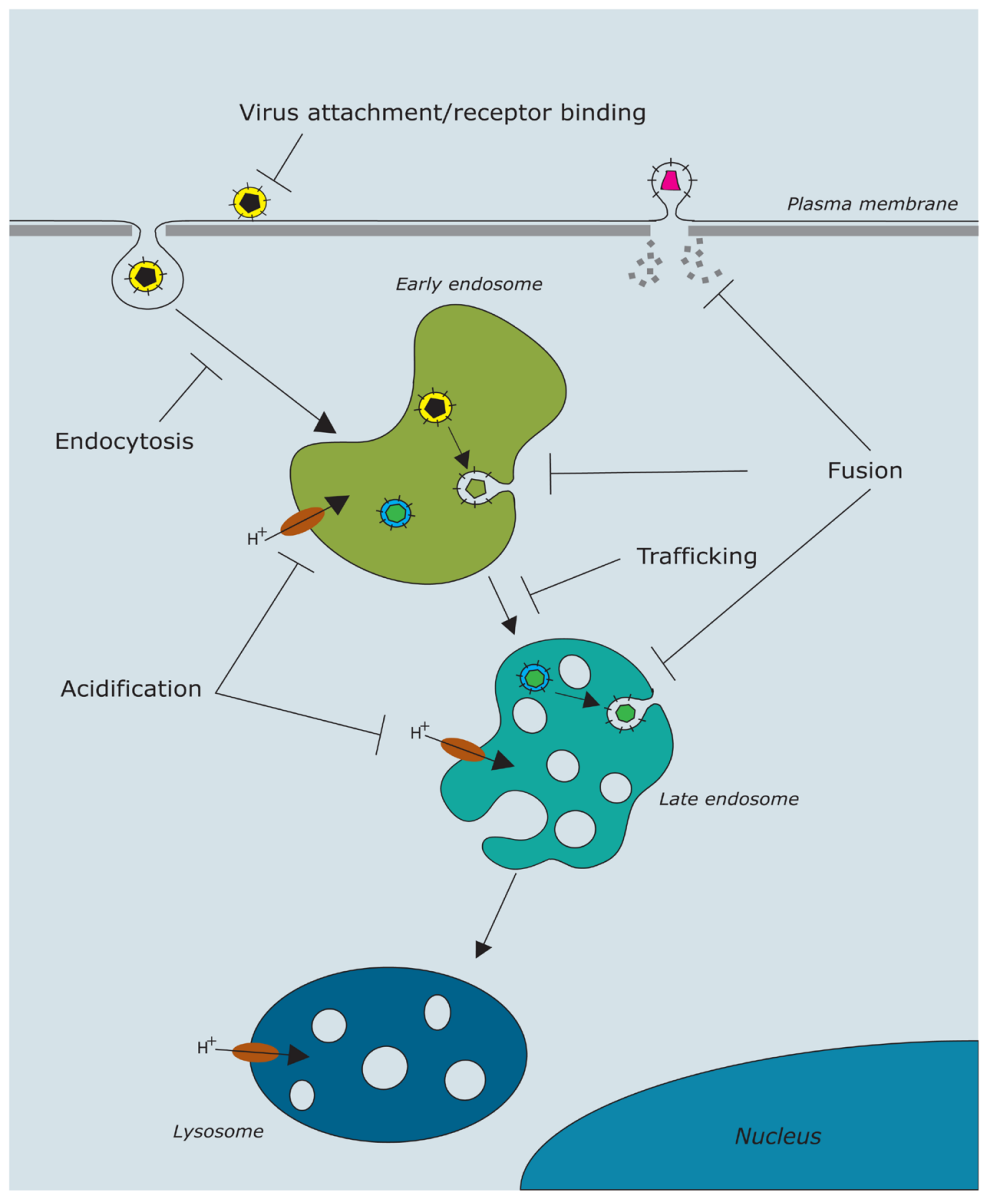
Schematic representing the main stages of viral entry. Processes inhibited by the antivirals described (attachment, receptor binding, endocytosis, trafficking, endosomal acidification, and fusion) are indicated.

In this review, we summarize the main therapeutic approaches that target different stages of viral entry ( Figure 1), either by inhibiting virus-specific interactions or by blocking conserved cellular mechanisms that viruses exploit to enter cells. This latter tactic is indicative of a change of focus for viral therapeutics, combining more traditional approaches directed at specific viruses with novel cell-targeted strategies with broad-spectrum potential.

## Inhibition of cell adhesion

The first step in viral entry is virus adhesion to the cell surface. Regardless of the requirement to engage specific receptors, viruses often use non-specific electrostatic interactions with negatively charged sugars to attach to a cell surface until they encounter a specific entry receptor. Negatively charged heparan sulfate proteoglycans (HSPGs) are expressed on the surface of most eukaryotic cells and have been shown to be required for the binding of many viruses, including human immunodeficiency virus (HIV), herpes simplex virus (HSV), human cytomegalovirus (HCMV), human papilloma virus (HPV), respiratory syncytial virus (RSV), and flaviviruses ^7– 10^. Inhibition of these interactions through the development of “decoy” particles mimicking these molecules (heparin, sulfated polysaccharides, or sulfonic acid-coated nanoparticle) can effectively prevent infection *in vitro*. However, limited efficacy is seen *in vivo*, possibly owing to the poor bioavailability of these formulations. While high-molecular-weight molecules with a high degree of sulfation have the highest antiviral activity *in vitro*, these compounds tend to bind to plasma proteins and have poor bioavailability ^11, 12^. Conversely, low-molecular-weight compounds have better bioavailability and are more effective *in vivo* ^13^ but are also associated with higher cytotoxicity, possibly owing to detergent effects on cellular membranes.

Virucidal activity may also be required to increase *in vivo* efficacy. Cagno *et al*. have shown that nanoparticles carrying long, flexible linkers mimicking HSPGs can simultaneously bind multiple sites on a virus and cause permanent distortion of the virion structure. This virucidal activity was sufficient to inhibit RSV infection in mice, suggesting that particles mimicking the negatively charged cellular surfaces to which viruses bind have the potential to be effective antivirals, as long as they include the capacity to inactivate viral infectivity ^14^.

It is important to note that sulfated polysaccharides are also known to have anticoagulant activity, but derivatives exist that do not show these properties *in vivo* at therapeutic doses (e.g. fucoidan, galactan, and xylomannan) ^12^.

Other molecules that target common cellular components also have the potential to interfere with attachment and to have broad-spectrum antiviral activity. Cyanovirin-N is a naturally occurring lectin that has been shown to inhibit the attachment of HIV ^15^ and Ebola virus (EBOV) to cells ^16^, most likely by binding to high-mannose oligosaccharides on the viral glycoproteins. The same mode of action may prevent infection by other viruses, though this remains to be tested, as does *in vivo* efficacy *.*

## Inhibition of receptor binding

### Cell surface receptors

While often involved in virus adhesion to cell surfaces, negatively charged sugars associated with cell surface glycoproteins and glycolipids can also act as virus receptors (e.g. sialic acid for influenza) or enable an interaction of sufficient strength to allow internalization of a virus by endocytosis, as may be the case for flaviviruses and filoviruses ^17, 18^. Both virus- and cell-targeted approaches have been studied to prevent the interaction between influenza hemagglutinin (HA) and sialic acids (SA) on glycoprotein and glycolipid receptors. Virus-targeted strategies include the use of protease inhibitors that block the processing of HA and peptides or small molecules that interfere with HA binding to SA. The most promising cell-targeted strategy is the attachment inhibitor DAS181, a recombinant protein made of a sialidase catalytic domain (which removes SA from influenza receptors) and a mucosal cell-surface anchoring sequence. This compound, highly potent *in vitro* (IC _50_ between 0.04 and 0.9 nM), has been shown to be non-toxic to cells and to be effective *in vivo* when administered pre- and post-exposure ^19^. A phase II trial showed reduction of viral load over 5 days’ administration at 10 mg/kg/day ^20^, and phase III trials are ongoing. A comprehensive overview of inhibitors of HA–SA interaction is presented by ^12, 21^.

For other cell-surface virus receptors, maraviroc (FDA approved in 2007) is currently the only drug that inhibits viral entry. Its target is CCR5, the major co-receptor required for HIV infection of CD4-positive cells ^22^. Maraviroc was developed by Pfizer through screening of their compound library and subsequent additional screens to improve activity and pharmacological properties of the initial hit compound. In combination with at least two additional anti-retroviral drugs, maraviroc is currently used in HIV-infected patients showing resistance to other compounds ^23^. While some limited development of resistance has been observed, the frequency of this is not clear ^22^ and is usually associated with multiple mutations in the HIV envelope protein that generally cause a significant decrease in viral fitness ^24^. This notion, that viral adaptations to overcome inhibitors acting through cellular targets can weaken the virus, is a potentially significant and interesting advantage of host-targeted antiviral strategies.

An alternative tactic is to inhibit the synthesis of cellular membrane glycoproteins that function as receptors for specific viruses. Cyclotriazadisulfonamide (CADA), a small-molecule inhibitor of HIV replication, has been found to specifically inhibit the synthesis of the HIV receptor CD4 by binding to the protein signal sequence in the Sec61 translocon during the co-translational insertion of the nascent protein into the ER membrane ^25^. Decreased levels of CD4 render potential host cells refractory to HIV-1 infection and presumably HIV-2, HHV6, and any other viruses that use CD4 as a receptor. Whether this approach is effective *in vivo* and whether it can be developed to inhibit the synthesis of a broader range of virus receptors remains to be seen (see 25 for a discussion of evidence for other compounds that inhibit the synthesis of specific cell-surface proteins).

Inhibiting the interaction of a virus with target cells is also one of the main modes of action of antibodies, and the identification and generation of such antibodies remain major strategies for antiviral immunotherapies. The development of small molecules that mimic the effect of antibodies and can be administered orally also has potential. Scientists from Janssen developed such an approach starting from the identification of a broadly neutralizing antibody, bnAbCR6261, that targets a conserved region in the stem of influenza HA. They then used a displacement screening strategy to identify small molecules that could bind to the same region as the antibody. Further medicinal chemistry on the most promising hit led to compound JNJ4796, a benzylpiperazine derivative able to inhibit influenza infection in 3D cultures of human bronchial epithelial cells and in a mouse model. Encouragingly, protection from lethal challenge was seen after oral administration in this latter model ^26^.

### Intracellular receptors

While most virus receptors are found at the cell surface, some viruses also interact with receptors inside endo-lysosomal compartments following their endocytosis. For example, EBOV uses the lysosomal protein Niemann Pick C-1 (NPC-1) ^18^, while Lassa fever virus has been shown to interact with LAMP1, another lysosomal protein ^27^. Antibody targeting of these interactions is complicated by the intracellular localization of the receptors. However, an exciting technology that has recently been expanded to viral infection is the development of bispecific antibodies. By linking the variable regions of an antibody recognizing the EBOV glycoprotein (GP) glycan cap (FV-M09) with the variable regions of antibodies recognizing either NPC-1 (mAb-548) or the NPC-1 binding region on GP (MR72), Wec *et al*. showed that this bispecific antibody bound to EBOV is internalized into endo-lysosomal compartments where it potently blocks Ebola interaction with its intracellular receptor ^28^. This strategy, already tested for HIV ^29^, has been shown to protect mice, even when administered 2 days after challenge with EBOV ^28^.

Small-molecule inhibitors of NPC-1 that accumulate in endosomal compartments like U18666A ^18^ and the piperazine-derivative 3.47 ^30^ also effectively block EBOV infection *in vitro*, the first by inducing cholesterol accumulation in the lysosomes and the latter by directly interacting with NPC-1.

### Non-enveloped viruses

Compounds also have been developed that selectively bind to non-enveloped virus capsid proteins to prevent interaction with cellular receptors or uncoating. One example is the enterovirus capsid inhibitor pocapavir ^31^, which has been tested as an investigational drug to treat neonatal viral sepsis ^32^ and has also been trialed against the spread of attenuated poliovirus from vaccinated individuals ^11^. Pocapavir and other capsid binders fit into a hydrophobic pocket in the viral particle, preventing conformational changes required for uncoating ^33^, but have the disadvantage of selecting for drug-resistant mutants. More recently, a conserved VP1–VP3 interprotomer interface in the viral capsid, critical for the conformational changes necessary for RNA release, has been proposed as an alternative druggable target. Compounds binding to this interface have been shown to be active against a larger number of enteroviruses and also against rhinoviruses, though their ability to induce resistant variants has yet to be investigated ^34^. Tryptophan dendrimers that target the 5-fold-axis of the enterovirus-A71 capsid have also been shown to have antiviral potential *in vitro* by preventing virus interaction with the (co-)receptors PSGL1 and heparan sulfate ^35^. ****

## Inhibition of internalization

For viruses that do not penetrate directly at the plasma membrane, endocytosis is an essential step in the entry process. Endocytosis can be blocked by compounds that interfere with signaling cascades necessary to activate endocytosis or that inhibit the endocytosis machinery directly. The PI3K-AKT signaling pathway, for instance, has been shown to be required by a variety of viruses ^36, 37^, and its inhibition can prevent infection *in vitro* ^6^. Similarly, vaccinia virus (VV) entry requires signaling through epidermal growth factor receptors (EGFR), serine/threonine kinases, protein kinase C, and p21-activated kinase 1 in addition to PI3K, and entry of at least some VV strains is sensitive to the tyrosine kinase inhibitor Genistein ^38^. However, studies of their effectiveness *in vivo* are limited. While there is evidence that preventing PI3K/AKT activation reduces pathogenesis in mice infected with the alphavirus Ross River, this is likely to be due to post-entry effects on virus replication and cellular metabolism ^37^.

A number of research compounds exist that block endocytosis directly, including PitStop, which inhibits the formation of clathrin-coated pits ^39^, as well as Dynasore ^40^ and the more potent derivative Dyngo-4a ^41^, which interfere with dynamin. While these compounds have been shown to inhibit the entry of various viruses *in vitro*, they have not been used therapeutically as antivirals. A study by Harper *et al*. showed that Dyngo-4a can inhibit botulinum neurotoxin type A endocytosis in neurons *in vitro*; however, *in vivo* administration only delayed the onset of symptoms by ~3 hours in 60% of infected mice. Encouragingly, animals treated with Dyngo-4a did not show signs of toxicity at the concentrations tested ^42^. Whether higher concentrations are required to see a stronger effect *in vivo,* whether these have higher toxicity (because of either inhibition of endocytosis or off-target effects), and whether virus infection is affected requires further investigation. Several other compounds including chlorpromazine, chloroquine, and Arbidol have also been shown to inhibit endocytosis and have an antiviral effect *in vitro* ^43, 44^, but efficacy *in vivo* has not yet been reported.

In a recent study, starting from phenotypic screening with representatives of different virus families, we identified two compounds, niclosamide and Tyrphostin A9, with very broad-spectrum antiviral activity, affecting different stages of virus replication, including endocytosis ^45^. The most likely mode of action is through dissipation of proton gradients and inhibition of ATP synthesis ^46– 48^, which impact on multiple cellular pathways. However, using endocytosis assays, we were able to show that CME of the alphavirus Semliki Forest virus and of fluorescently labeled transferrin was significantly reduced by both compounds ^45^. While the pharmacokinetic properties of niclosamide prevent its accumulation in the blood ^49^, Tyrphostin A9 had a better profile and showed some ability to inhibit Zika virus infection in a mouse model ^45^. Tyrphostin A9 is also a general tyrosine kinase inhibitor and is likely to affect a variety of kinases involved in viral life cycles. Interestingly, Tyrphostin A23 has also been shown to inhibit CME by preventing the interaction between receptor tyrosine-based endocytic signals and the mu2 subunit of the AP-2 adaptor complex ^46^. Although the chemical properties of Tyrphostin A9 make it unlikely to become a drug, this work provided proof of concept that entry inhibitors with broad-spectrum antiviral activity *in vitro* can also exhibit antiviral activity *in vivo*.

## Inhibition of fusion

For enveloped viruses, fusion is the next necessary step that allows a virus to release its genome into the cytoplasm. Fusion is mediated by virally encoded envelope glycoproteins displayed on the virion surface. Viruses require cellular cues to induce conformational changes in these envelope glycoproteins to initiate fusion with cellular membranes, in some cases the plasma membrane, and, for many viruses, endosomal membranes. For some viruses that fuse at the cell surface, such as HIV, receptor engagement provides the cue that initiates the envelope glycoprotein conformational changes and fusion ^50^. But for many viruses a common cue is the acidic pH of endosomal environments ^1, 3, 51^. Weak bases, including the antimalarial drug chloroquine, and carboxylic ionophores such as monensin, can inhibit endosomal acidification and block infection by a wide range of viruses *in vitro* ^1, 52, 53^, but, with the exception of amantadine, few studies have tested their efficacy *in vivo.* The weak base amantadine and some amantadine analogues have been used against influenza virus infection ^54^, but their activity is directed through inhibition of the viral M2 protein, rather than inhibition of endosomal acidification, and viruses rapidly evolve resistance ^55^.

An alternative cue to low pH is proteolytic cleavage of envelope proteins by endosomal proteases, as used by filoviruses, such as EBOV, and coronaviruses. For EBOV, proteolytic cleavage by endo-lysosomal cathepsins is essential to generate a form of the envelope glycoprotein that can bind NPC-1 ^56^, and inhibitors of these proteases can effectively abrogate EBOV infection, although none of these compounds is currently approved for use in humans and selectivity remains an issue. Recently, a role for the two-pore calcium channel 2 (TPC2) in EBOV infection was shown. While the exact role of TPC2 is not yet clear, it is likely to control cues in the endo-lysosomal environment necessary for EBOV fusion ^57^. Interestingly, small molecules that target TPC2 inhibit EBOV infection both *in vitro* and *in vivo* ^57– 59^. These TPC2 antagonists, including tetrandrine, also inhibit MERS coronaviruses ^60^. Similarly, apilimod, a compound that targets a phosphatidylinositol-3-phosphate 5-kinase (PIKfyve) involved in endosomal trafficking, also inhibits EBOV infection by blocking the transport of Ebola particles into NPC-1-containing endo-lysosomes ^61^. Whether these compounds act on other viruses that penetrate from late endosomes remains unclear. Elevation of endosomal potassium may also provide cues that activate the fusion proteins of some bunyaviruses and influenza viruses, and infection can be restricted by compounds that inhibit endosomal potassium channels ^62^. Together, these studies indicate that combining perturbants of the endocytic system might offer an effective approach to inhibiting a range of different viruses.

Some virus-specific fusion inhibitors also exist. Enfuvirtide, used in combination with other anti-retroviral therapies for the treatment of HIV, is a peptide that binds the heptad repeat region 2 in the gp41 subunit of the HIV envelope glycoprotein and prevents formation of the so-called stable six-helix bundle that is crucial for membrane fusion ^63^. Fusion inhibitors that stabilize metastable conformations of the RSV fusion protein have also been developed ^64, 65^. Though none of these is currently approved for use, a number are in clinical trials. Peptides that block the fusion of other paramyxoviruses, including measles, Nipah, and Hendra viruses, by interfering with the viral envelope proteins have also been developed and shown to have antiviral activity *in vivo* ^57, 66, 67^. A similar approach using peptides or small molecules has also shown promise against flaviviruses (West Nile and dengue viruses) ^68, 69^.

An alternative approach to inhibiting fusion is to interfere with the biophysical properties of membranes through compounds that intercalate into cellular membranes and in so doing alter their rigidity or curvature. This can be achieved with certain lipids in tissue culture systems ^70, 71^. A similar effect may underlie the action of cellular proteins called interferon-inducible transmembrane proteins (IFITMs). Although the exact mode of action of these proteins (three in humans, IFITM 1–3) remains unclear, their effectiveness against an extraordinary variety of viruses suggests that interfering with the composition/biophysical properties of cellular membranes involved in virus entry can be a potent broad-spectrum antiviral strategy ^64, 72, 73^. However, recent work by Buchrieser *et al*. indicates that IFITM expression can interfere with placental development by inhibiting syncytin-mediated syncytiotrophoblast formation, indicating that these proteins can interfere in cellular fusion reactions ^74^. Whether they impact on other cell–cell fusion events or intracellular membrane fusion processes remains to be established.

Using phenotypic screens, we and others have identified a number of compounds that, because of their biophysical properties, are likely to intercalate into the endosomal membranes and prevent fusion ^45, 75^. This is possibly the mode of action of selective estrogen receptor modulators (SERMs) and similar amphipathic compounds, including amiodarone ^76, 77^ and amodiaquine ^78^, although it is possible that these compounds may also alter endosomal pH. However, the concentrations required to achieve similar antiviral effects *in vivo* are likely to be high, and the risk of side effects significant.

Interfering with viral rather than cellular membranes is also a promising broad-spectrum antiviral approach for enveloped viruses. LJ001 is a small molecule which oxidizes the unsaturated phospholipids of both cellular and viral membranes ^79^. This is likely to alter membrane curvature and fluidity, preventing fusion. Interestingly, while affecting both cellular and viral membranes, only the latter are significantly affected, most likely because the cellular membrane can rapidly replace damaged lipids, a property that viruses do not share ^80^. This makes LJ001 effective *in vitro* against a wide variety of viruses including influenza, and with very limited toxicity. Unfortunately, little effectiveness was seen *in vivo*, owing to a short half-life (~4 hours) and low concentration of the compound in serum. Furthermore, LJ001 needs light to activate, which limits its application *in vivo*. Nevertheless, analogues with improved stability and pharmacokinetics are being developed ^79^.

## Conclusions and future outlook

Host cell entry is a crucial step for most viruses, and preventing entry provides a clear early opportunity to control infection. As viruses use just a few conserved cellular pathways for entry, targeting cellular proteins rather than viral proteins has emerged as a promising antiviral strategy. Indeed, many of the approaches that have been investigated in the past few years target host rather than viral components, even when the target is highly selective for one specific virus, as is the case for maraviroc ^22^. This is in line with a recent trend in the development of antiviral strategies ^51, 81^. To date, small-molecule interference with viral infection has tended to focus on specific viruses and viral proteins. For a few viruses, HIV, HCV, and herpes, for example, this approach has been very effective. In the case of HIV at least, combinations of compounds targeting key viral enzymes essential for completion of the viral life cycle (e.g. HIV *Pol-*encoded reverse transcriptase, integrase, and protease) control virus replication in infected patients, substantially improve longevity and life quality, and are now being adopted as pre- and post-exposure prophylactics to prevent virus infection ^82, 83^. Similarly, nucleoside analogues and protease inhibitors active against HCV have the potential to cure what would otherwise be a lifelong chronic infection often resulting in cirrhosis and liver cancer and only treatable by liver transplantation ^84^. Though highly successful, these drugs are limited to the treatment of single viruses and are prone to viral resistance mutations rendering them ineffective.

Conversely, very few drugs are available for use against the majority of other viruses. This is a major concern given the increased emergence (or re-emergence) of new pathogens, often from zoonotic infections (e.g. SARS and MERS), and the enlarged geographical distribution of insect-vectored viruses caused by environmental changes (e.g. Zika and dengue ^85^). Under these circumstances, targeting host cell pathways as opposed to viral proteins is a viable option for controlling infection. One argument against this approach is the potential toxicity of targeting host cell pathways. Toxicity could be due to the inhibition of critical cellular pathways as well as to off-target effects. However, most of the virus infections that might benefit from this approach establish acute infections for which drug administration could be as short as a few days. Moreover, most drugs currently on the market against a range of diseases target host cell pathways with limited, or acceptable, side effects. Another argument is that virus-targeted antivirals seem to be more potent than cell-targeted ones. This concern is counter-balanced by the potential for broad-spectrum effectiveness of many cell-targeted approaches, which may be critical to rapidly contain emerging viruses. Because of lower potency, these compounds might not stop virus infection completely, but several studies indicate that, in general, any capacity to reduce the viral load gives patients a better chance of establishing effective immune control ^86– 89^.

As with the application of anti-retroviral therapies, compounds targeting entry and replication could be used in combination; for example, compounds that inhibit CME could be administered together with compounds that inhibit macropinocytosis and/or endosomal acidification in order to broaden the range of viruses affected and/or increase the effectiveness of the treatment. Although we have focused on virus entry, targeting other cellular pathways, such as ER-associated glycoprotein synthesis, also offers exciting potential for broad-spectrum antiviral development that could broaden potential combination therapies ^90^. This strategy could also reduce toxicity by allowing the use of lower concentrations of each component in complex formulations.

While many successful approaches have been described *in vitro*, an outstanding question is whether they are also effective *in vivo*. In particular for host-targeted approaches, it will be important to determine safe thresholds that separate an effective decrease in viral load from toxicity. The limited *in vivo* efficacy of many treatments to date may be due to the pharmacokinetics and bioavailability of the compounds, or possibly to the limited range of conditions that are generally tested *in vivo* due to the costs, logistics, and restrictions on animal studies. Limited availability of adequate small models that recapitulate disease also contributes to the problem. While more work remains to be done, the approval of drugs like maraviroc and enfuvirtide suggests that targeting viral entry is a promising antiviral strategy warranting further investigation and the integration of different disciplines, from virology to medicinal chemistry.
